# Circadian Corticosterone Profile in Laying Hens (*Gallus gallus domesticus*)

**DOI:** 10.3390/ani14060873

**Published:** 2024-03-12

**Authors:** Theresa Hillebrecht, Rüdiger Korbel, Monika Rinder, Manfred Gahr

**Affiliations:** 1 Clinic for Birds, Small Mammals, Reptiles and Ornamental Fish, Ludwig-Maximilians-Universität München, 85764 Oberschleißheim, Germany; vorstandsassistenz@vogelklinik.vetmed.uni-muenchen.de (R.K.); monika.rinder@lmu.de (M.R.); 2 Max Planck Institute for Biological Intelligence, 82319 Starnberg, Germany; communications@bi.mpg.de

**Keywords:** circadian, chickens, corticosterone, physiology, chicken social interactions

## Abstract

**Simple Summary:**

Blood corticosterone concentrations are frequently used to evaluate stress responses in birds. Therefore, knowledge about physiological concentrations and daily fluctuations of this “stress hormone” is necessary. Corticosterone fluctuations in socially acting chickens are largely unknown, and we aimed to verify the presence of a daily rhythm. Therefore, blood samples were taken at intervals of four hours via a vein catheter over a period of three days from a total of 12 laying hens housed in groups of four in an enriched environment while video and audio recording their social interactions. Prior to the experimental phase, all hens were medically trained via adaption to repeated handling to reduce their stress reaction. In most hens, corticosterone showed a circadian course with two elevations per day. Statistical analysis revealed a significant peak during daytime (between 12:00 p.m. and 04:00 p.m.), and a tendency for a second peak during the night (12:00 a.m.). Further studies are necessary to elucidate the underlying control mechanisms, light and seasonal influences as well the function of the nightly corticosterone peak.

**Abstract:**

Measurement of blood corticosterone concentrations has been established as an indicator for assessment of acute distress. Therefore, knowledge on physiological fluctuations is required, but previous studies allow little conclusion on daily fluctuations in domestic chickens (*Gallus gallus domesticus*). To verify the presence of a circadian corticosterone rhythm in socialized chickens, blood samples were taken at four-hour intervals from 12 laying hens kept in groups of four over three days, each. Prior to experiments, hens were adapted to repeated handling for stress reduction. Corticosterone concentration was determined using radioimmunoassay. Blood sampling time and duration were recorded, and audio and video recordings were analyzed to assess the impact of behavior on corticosterone concentrations. Despite individual fluctuations, most hens showed a circadian course with two peaks per day. Statistics revealed a significant peak during the day (between 12:00 p.m. and 04:00 p.m.) and a tendency for a second peak at night (12:00 a.m.). The daily corticosterone peak was not explained by daytime social stress and needs to be seen as an endophenotype. The role of nightly corticosterone production has to be investigated in further studies. There might be a relation between corticosterone and reproduction since the only hen not showing peaks was not laying eggs.

## 1. Introduction

In birds, corticosterone (CORT) is the primary glucocorticoid besides cortisol, which is secreted in much lower concentrations [1]. CORT has been addressed in several studies as the relevant hormone for the detection of acute and chronic stress in birds [2,3,4,5,6,7], which requires knowledge on physiological homeostasis and on indicators useful for stress response diagnostics. Exposure to stimuli may result in a threat to homoeostasis, called stress, whereby a differentiation must be made between sustress, eustress and distress [8]. Sustress, also called inadequate stress, describes a state of homeostasis in which stimuli are too weak to challenge an organism. Eustress has been defined as positive stress which puts a strain on the body but enables it to solve difficult tasks. In contrast, distress describes negative stress which challenges the organism in the strongest way with high levels of stressors. Distress is able to cause a severe stress response and affect health [8]. Distress may translate into patterns of behavior that are not always recognized by owners [9,10,11,12]. In the chicken, this might include obvious signs such as beak breathing, polypnea and aggressive behavior towards people and/or conspecifics but also behavioral patterns such as feather plucking at flock members [9,10,11,12].

Physiologically, CORT influences various functions in the organism, including carbohydrate, lipid and protein metabolism, the urinary system and the immune system [7,13,14]. In addition, CORT shows an increase at the time of oviposition, although the underlying mechanisms are not yet fully understood [15].

Blood CORT may fluctuate dependent on environmental and endocrine stimuli over time, leading to individual variations. It reflects current fluctuations in the blood with a change in hormone concentrations about 45 s to three minutes after exposure to an acute stressor [16,17,18]. Seasonal conditions such as daylength, brightness, temperature, humidity or reproduction state as well as other external factors like stocking density, suboptimal housing conditions, inappropriate handling/transport and social interactions have a clear impact on animals’ condition [19]. Furthermore, individuals may vary in their susceptibility to stressors due to escape-reflex-oriented behavior or due to individual physiology as well as the amount of corticosteroid binding globulin (CBG) in the blood [20,21,22,23,24,25]. When determining plasma CORT using available assays, it is important to consider that the measures do not reflect unbound, biologically active CORT but show total CORT concentrations which depend in part on seasonal and age-dependent fluctuations in the CBG’s binding affinity [20,22].

Repeated measurements of hormone concentrations in the same individual during a day, which are essential for behavioral and endocrine studies to establish a reliable hormone profile, are very rarely performed. In the rare attempts for circadian CORT profiles, different individual chickens were sampled at different times, each, [26,27] or in experiments in which the same animals were sampled several times, social interactions within a flock were not considered or the animals were kept individually [28,29,30,31,32].

We therefore aimed to describe a profile of CORT concentrations over a course of several days in domestic chickens (*Gallus gallus domesticus*) kept in small groups. Since we expected that exposure to unexpected stressors and social interactions, especially aggressive behavior, could influence the CORT values, we recorded and analyzed behavior and sounds of the chicken during the complete experimental procedure.

## 2. Materials and Methods

### 2.1. Animals and Experimental Procedure

The present study was planned as a feasibility study to keep the number of animals required as low as possible in line with the 3R principle (Replacement, Reduction, Refinement) [33,34]. The experiments were conducted in accordance with the German animal welfare regulations and under permission of the German authorities (reference number ROB 55.2-2532.Vet_02-20-161).

Commercially reared Lohmann Brown classic laying hens (n = 12), aged 26–30 weeks at a minimum body weight of 1.5 kg, were used. Out of 12 hens, 11 reached sexual maturity prior the experimental phase. Only hen ten did not reach sexual maturity until the end of the experiments which was indicated by the first oviposition about two weeks after the experimental phase (eggs differed in size and color). All animals were clinically and parasitologically monitored by veterinarians. Only clinically healthy animals were used for the experiments. They were kept in groups of four in 11.21 m^2^ aviaries filled with straw pellet litter and enriched according to German (TierSchNutztV, Section 3, §§ 13, 13a) and European legislation (Directive 1999/74/EG; Directive 2010/63/EU) [35,36] for poultry and experimental animals and received water and feed (layer feed Legemehl Premium and mixed grain feed Vogelkörner Premium, Mifuma, Mannheim, Germany) ad libitum. Stocking density was 0.36 hens/m^2^, and the aviaries used provided an enriched environment like small backyard poultry husbandries. Protection from infectious diseases was achieved by negative pressure ventilation, access restriction and hygiene sluices. A pecking stone (Pickblock, Crystalyx Products, Münster, Germany), alfalfa hay bales (Compact Luzerne, Hartog, Lambertschaag, The Netherlands) and fresh fruits or vegetables were offered as enrichment on a daily basis. The hens were exposed to the natural daylight due to UV-transmitting windows considering avian specific UV-perception. The experiments were conducted successively, resulting in different light regimes (light–darkness) of 15L:9D (July, group one), 14L:10D (August, group two) and 11L:13D (September/October, group three). Temperature and relative humidity averaged 22.6 °C and 52%, respectively, during experiments.

Daily care, training and the whole experimental procedure were performed by the same person. Prior to the experimental phase, all hens were trained on a daily basis with rewards like mealworms, corn or grain mix to get used to being handled and to wear High-Visual chicken jackets (Yellow High-Vis Chicken Jacket, Omlet Ltd., Banbury, England) for at least two weeks [37,38]. Handling in the pre-experimental phase included repeated catching and holding the hens in the arm until beak breathing, polypnea or kicking as a sign of distress did not occur anymore. Adaptation to handling rapidly set in within approximately two weeks. After no distress associated behavior as described above during handling was displayed anymore, training with High-Visual chicken jackets was started. Depending on the individual behavior, the time to wear a jacket was slowly increased up to 5 h, starting with a minimum of two min. Mock bleeding was not performed in the training phase. Experimental procedure was started when all hens displayed normal behavior while wearing the High-Visual chicken jackets, assuming an adaption to experimental setting, and thus, non-elevated CORT concentrations [38].

A total of six test series with three groups of four hens were performed. In each test series, two hens of a group were blood sampled every four hours over a period of three days via venous catheter (Vasofix Braunüle 22 G, 25 mm, blue, B. Braun Melsungen AG, Melsungen, Germany), minimizing the time between the catch of the individuals as much as possible because all samplings were performed successively. The experiment started at day one at 10:00 a.m. with insertion of venous catheter in order to reduce the length of catheterization and to lower the risk of potential medical complications (e.g., traumatization of the venous endothelium), followed by the first of 18 samplings at 12:00 p.m. at day one, and taken until day four at 09:00 a.m. which depicted a period of three days (71 h) (Table 1). Venous catheters were inserted into the ulnar vein under isoflurane inhalation anesthesia (5 Vol. % isoflurane for induction and 2.5 Vol. % isoflurane for maintenance) with preemptive analgesia using 0.5 mg/kg meloxicam (Metacam^®^ 5 mg/mL solution for injection in dogs and cats, Boehringer Ingelheim Vetmedica GmbH, Ingelheim am Rhein, Germany) i. m. 0.5 h pre surgery. A 3% citrate buffer solution was used as anticoagulant and blocking solution for the catheter. The catheter was closed with an obturator (obturator for Vasofix, G 22 × 25 mm, blue, B. Braun Melsungen AG).

For the next test series, the remaining untested two hens of the same group were used. This procedure was used for the other two groups until all 12 hens were sampled 18 times (Supplementary Material Table S1). A blood volume of 0.5 to 0.8 mL was taken during each drawing resulting in a total volume of up to a maximum of 14 mL for each hen during the sampling period (<1% of body weight).

In order not to disturb the hens more than necessary during the experimental phase, the aviary was only entered for blood sampling or feeding. When entering the aviary, the laying nests with inserts of synthetic turf (polyethylene) or other preferred places such as the top of the metal ventilation system were checked for eggs and eggs were collected. Depending on the four-hour time slot when the eggs were collected, one could assess the time point when oviposition happened. The audio or video recordings gave further hints, such as a sound of an egg falling on ground (laying nest or ventilation box) or a hen staying for a long time in the laying nest.

### 2.2. Blood Sampling and Corticosterone Measurement

Blood samples were transferred to a heparin sample tube (1.3 mL micro sample tube, PE soft stopper, lithium heparin, SARSTEDT AG & Co. KG, Nümbrecht, Germany) and centrifuged within 5 min after collection for three minutes at 3500 g. Plasma was stored at −20 °C until analysis. CORT concentrations in plasma samples were measured using a radioimmunoassay (RIA) following a protocol previously described by Goymann et al. [39] using a liquid scintillation counter (Beckman Coulter LS6500 Multi-Purpose Scintillation Counter, Brea, CA, USA). The total amount of 216 plasma samples was processed in 3 batches between August and November 2021.

The time between capture and blood drawing was recorded in order to be able to evaluate a possible influence of a stress reaction triggered by capture and fixation of the chickens. The time between capture and blood sampling in each test animal averaged 2.5 min, with a minimum of 1 min and a maximum of 7 min (Supplementary Material Table S2). RIAs were performed in three batches with detection limits of 3.15–3.63 pg/mL. Measurement precision of the three RIA batches was good with an intra-assay coefficient of variation (CV) of 5.5–7.9%, inter-assay CV of 7.8%, intra-extraction CV of 4.7–7.1% and inter-extraction CV of 6.7%.

### 2.3. Behavioral Analysis

During each of the six test series, behavior and sounds of the animals were monitored with a surveillance camera (GeoVision, model GV-FER5701) fixed at the ceiling of the aviary and special microphones (in house product, Max Planck Institute for Biological Intelligence, Seewiesen, Germany) attached on the backs of the hens, covered by High-Visual chicken jackets to prevent pecking of the microphones. All test series took place one after the other in the same aviary. Therefore, each group of chickens was transferred to this room at least one week prior to test series for adaption to the new aviary. Recordings of video and audio files took place between 21 July 2021 and 09 October 2021. The analysis of video and audio files was conducted using the free software Audacity^®^ (Version 2.4.2) and GeoVision Multicam Surveillance System (Version 8.5.7.0) using the ViewLog function and parallel auditory and visual evaluation. Around 426 h of microphone (wav files) and video recordings (avi files) were obtained during the experimental phase. All audio tracks were visualized as spectrograms and the sounds simultaneously analyzed. Suspicious vocalizations were checked in the corresponding video records. Since dominance behavior was not always associated with vocalizations, full length of the video recordings was analyzed for such a behavior. The microphones failed to record sounds during the second test series; thus, from the third test series onward, an additional external microphone was placed in the aviary (Supplementary Material Table S1). Further, some events could not be visually assigned to the vocalizations because the chickens were sitting in a blind spot, such as under the ventilation or in the laying nest. These events were excluded from evaluation for physiological or distress-related CORT concentrations and mentioned separately. The same valuation was used for signs of distress occurring > 30 min prior sampling.

Chickens are known to produce a large amount of different call types [40,41,42,43,44,45,46,47,48,49], for which characteristic spectrograms, obtained during our experiments, are shown in Supplementary Material Figure S1. The sound spectrograms were particularly searched for short (0.33 s) and long (1 s) screams as a possible sign for discomfort or distress in the hens [48]. The distinction between distress calls and other call patterns was made according to the definition of Marx et a. in 2001 [50].

Aggressive behavior was defined as short attacks or fighting with serious harm caused to flock mates, e.g., to establish and maintain pecking hierarchy, to secure access to feed/water, to defend territory or due to stress [12]. Whereas dominant behavior was defined as threatening or pecking without harming flock mates, followed by immediate avoidance and subordination of the other hens without fighting [12].

### 2.4. Statistical Analyses

Descriptive statistics were applied to the individual animals and sampling times using R Studio (R version 4.1.3 (2022-03-10), Rstudio Team (2022). Rstudio: Integrated Development Environment for R. Rstudio, PBC, Boston, MA URL http://www.rstudio.com/, accessed on 10 March 2022). Shapiro–Wilk test and quantile–quantile plots were used for testing for normal distribution. Differences between hens’ hormone values were analyzed using the Kruskal–Wallis Test. For statistical analysis of the CORT values (n = 216), the first measurement of all hens was excluded, as there might be an influence of the anesthesia and transport distress shortly before the first blood sampling (n = 204). Studies in mice and rats have reported elevated CORT concentrations up to four hours after isoflurane anesthesia [51,52].

Linear mixed effects models are an efficient tool for analyzing complex data sets with repeated measurements and involve fixed factors as well as possible influences by random effects on the results. Robust regression of these allows the data sets to contain outliers as it reduces the weight of them [53]. Therefore, a robust variant of linear mixed effects model was used for our data set to describe the relationship between sampling time points as the fixed factor, hormone values as the dependent variable and the 12 hens as the random factor. A correction factor was not applied in order not to miss any effects. Time points as variables were dependent as measurements were repeated over three days in each animal (Supplementary Material Table S3), yet the single animals were independent variables. We did not expect any effects of age, since the age gap was only four weeks and our primary aim was the investigation of circadian CORT patterns in the blood. Therefore, we focused on the effect of the six sampling time points on CORT concentrations.

Estimated marginal means with a confidence level of 0.95 was applied as a post hoc test to describe differences between time point mean hormone concentrations. For evaluation of the post hoc test results, we used conventional thresholds for P-value interpretation as described before [54].

## 3. Results

### 3.1. Behaviour and Sounds

In our experiment, no aggression associated with pecking-related injury amongst flock mates was observed. Dominant behavior was apparent in all groups. Signs of dominance manifested in pecking head/comb/feathers during the day without harming, chasing hens away from food/water or out of the individual comfort zone and pecking during perching.

The number of experienced dominant behaviors which were accompanied by vocalizations varied strongly between individuals of the groups (Table 2).

E.g., hen two of group 1 was pecked 1 to 26 times per day and chased up to 28 times during the first test series. In the second test series, pecking frequency decreased to none to 12 times a day, but chasing remained high with 1 to 20 incidents. Hen one of this group was never recorded being pecked or chased, hen three was only pecked twice in the whole experiment, and hen four experienced one to four pecking incidents a day and was chased once during the experiment. In the second group, hen five was exposed to the most dominance gestures, with none to seven daily pecking incidents and being chased a maximum of two times. Hens six and eight were only pecked two times in both test series of group two, and hen seven was pecked three times and chased once during the experimental phase. In groups one and two, dominant behavior decreased over time. The animals of the third group showed the least dominant behavior, with hen nine being pecked three times during the experiment, hens eleven and twelve each being exposed to one pecking incident and hen ten not being exposed to any dominance behavior.

Hens were not harmed by dominance behavior during the experiments. Frequently, threatening or attacking was not accompanied with calls. In only a few cases, hens screamed as a reaction to being pecked or when being picked up for blood sampling, especially during the night. These events were assessed as distress (Table 2 and Supplementary Material Table S2).

All hens except one laid a single egg per day (for hen ten, no oviposition was observed before or during the experiments). This hen started laying eggs about two weeks after the end of the experimental phase. These eggs had a lighter shell color than the eggs of the other hens and were much smaller. The other three hens of group three were observed during oviposition prior to the experiment, and three eggs per day were found in the laying nests, likewise during the experiment. However, ovipositions could not always be recognized in the video recordings and could not be assigned to the individual hens, as most of them had very similar plumage color and all of them wore identical High-Visual chicken jackets. About half of the ovipositions were suspected based on behavior like staying in the laying nest for a longer time or audio recordings revealing sounds of an egg falling on hard ground, related to four-hour time slots where eggs were collected. Oviposition times were between 06:14 a.m. and 04:56 p.m.

### 3.2. CORT Concentrations

When CORT concentrations were analyzed over time, the first measurement was significantly higher than the following ones in eight out of twelve hens, with hen two showing the highest value of all animals (37,068 pg/mL) (Supplementary Material Table S4). The increased values during the first measurement point can be explained by the special conditions of this first sampling. The hens received permanent catheters before the first sampling. For this purpose, they were transported to the nearby surgical suite and underwent isoflurane inhalation anesthesia two hours before the first sampling. The increased CORT values can thus be attributed to the two-minute transport and to the subsequent isoflurane inhalation anesthesia and recovery phase. Studies in mice and rats have reported elevated CORT concentrations up to four hours after isoflurane anesthesia [51,52]. In order to avoid distortion of the circadian hormone profile, the first measurements were excluded from further statistics.

Excluding the concentrations of the first sampling, minimum CORT values of all hens ranged between 479.3 pg/mL and 1237.3 pg/mL, while maximum values were between 2319.4 pg/mL and 9958.9 pg/mL. The hens’ mean CORT concentrations ranged between 885.7 ± 716.4 pg/mL and 4532.7 ± 2592.4 pg/mL (Supplementary Material Table S5). The data were not normally distributed (Supplementary Material Table S6 and Figure S2). Hens’ distribution of CORT values differed between the individuals (Supplementary Material Figure S3).

**Figure 1:**
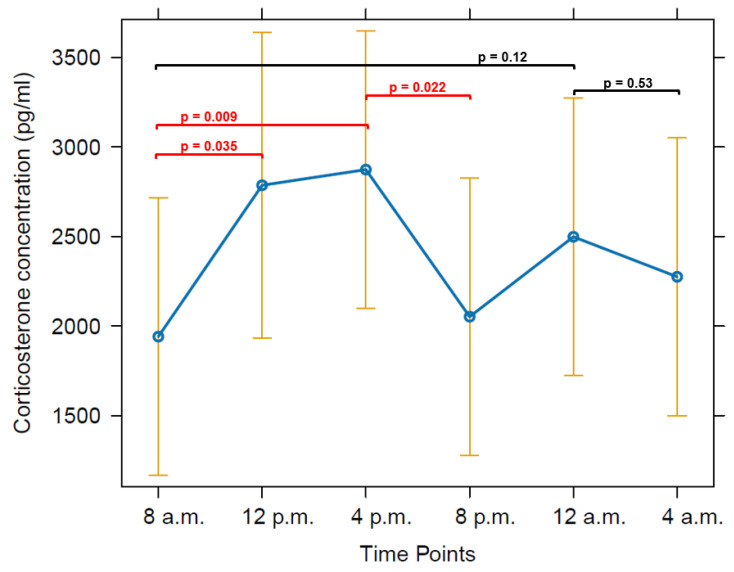
Effect plot for diurnal corticosterone concentrations of all hens (exclusive data from first time point as they might be elevated due to transport stress and isoflurane anesthesia). It pictures the effect of the sampling time points as a fixed factor on the estimated marginal means (emmeans) of CORT values as the dependent variable, indicating a pronounced peak between 12:00 p.m. and 04:00 p.m. as well as a tendency for a second peak at midnight. Lower and upper confidence levels are given in orange. Significant differences between time points (contrasts of emmeans) are displayed in red with according *p*-values, non-significant differences for the peak tendency at midnight are displayed in black with only upper and lower *p*-values.

Ten hens (all but hens two and ten) showed CORT profiles with several noticeable peaks over the course of three days. Hen two did not display many peaks over the three days, often just one peak per day. Hen ten showed the lowest CORT concentrations of all hens without any distinct peaks. Most animals showed the highest CORT concentrations at noon (12:00 p.m. or 04:00 p.m.) and at night (12:00 a.m.). This was confirmed by a robust linear mixed effects model revealing highest mean CORT concentrations between 12:00 p.m. and 04:00 p.m. (*p* < 0.05) (Figure 1, Supplementary Material Tables S7–S9). The nightly concentrations might indicate a second peak; however, this potential peak was statistically not significant (0.12 < *p* < 0.53) when a robust linear mixed effects model was applied to all hens (Figure 1, Supplementary Material Table S9). These results were confirmed by comparing the estimated marginal means of the CORT concentrations (Supplementary Material Tables S8 and S9).

When evaluating each group separately, the hens of group one (hens one to four) showed significant, highest mean CORT concentrations at 04:00 p.m. (*p* < 0.05) and a lower second, non-significant peak at 12:00 a.m. (0.07 < *p* < 0.87) (Supplementary Material Figure S4). The effect model plot for hens of group two (hens five to eight) showed a continuous rise from 08:00 p.m. to 08:00 a.m., followed by a decline without significant elevations (Supplementary Material Figure S5). Group three (hens nine to twelve) had a significant elevation at 12:00 p.m. (*p* < 0.05). Thereafter, CORT concentrations decreased until 08:00 a.m. (Supplementary Material Figure S6).

**Figure 2:**
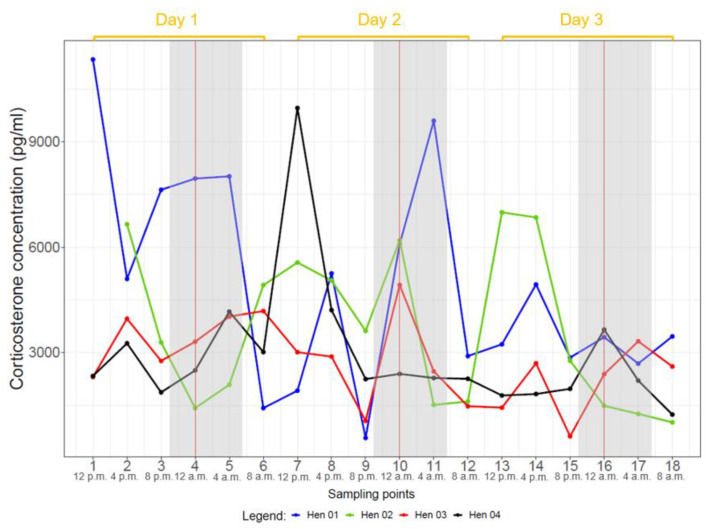
Plasma CORT profiles of hens of group 1 (hen 1 to hen 4) during 3 days. Samples of each hen were taken at 18 sampling points at intervals of 4 h. Grey background represents dark phase and the vertical red line represents midnight (12:00 a.m.).

**Figure 3:**
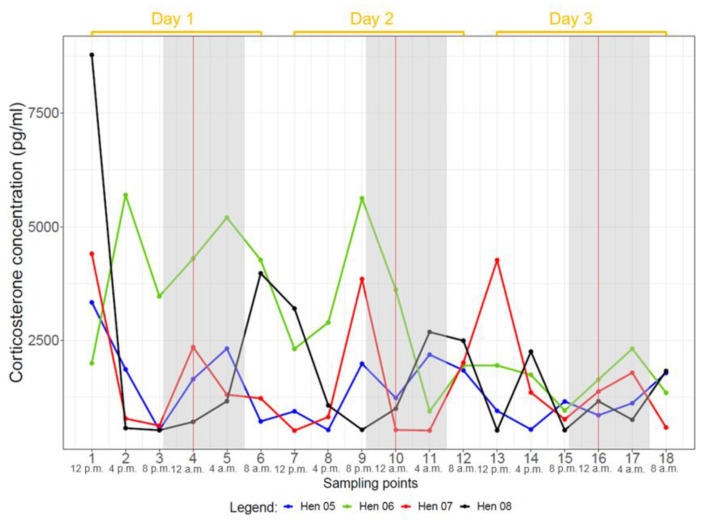
Plasma CORT profiles of hens of group 2 (hen 5 to hen 8) during 3 days. Samples of each hen were taken at 18 sampling points at intervals of 4 h. Grey background represents dark phase and the vertical red line represents midnight (12:00 a.m.).

**Figure 4:**
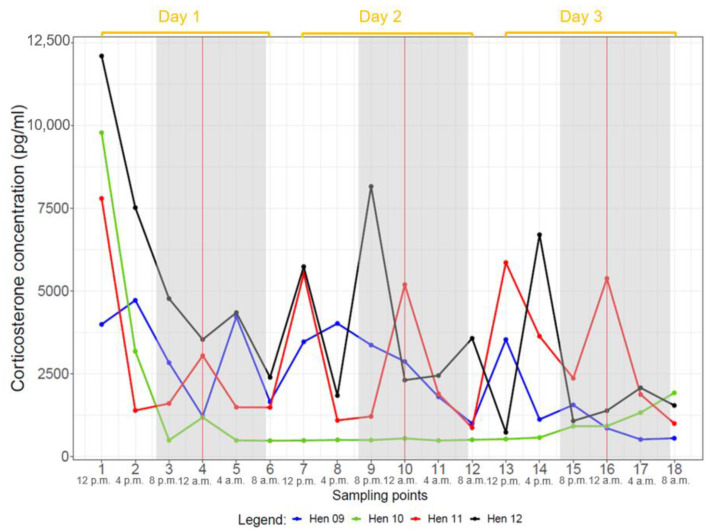
Plasma CORT profiles of hens of group 3 (hen 9 to hen 12) during 3 days. Samples of each hen were taken at 18 sampling points in intervals of 4 h. Grey background represents dark phase and the vertical red line represents midnight (12:00 a.m.).

The intervals between individual peaks differed depending on the individual animals but were mainly 12 h (Supplementary Material Table S2). However, single hens had intervals of 8 h (e.g., hens five and twelve), 16 h (e.g., hen three) or 20 h (e.g., hen eight), which caused time shifts in the peaks for the following days (Figure 2, Figure 3 and Figure 4, Supplementary Material Table S2).

The mean time span between catching and blood sampling was 2.5 min and was not correlated with CORT concentrations (Supplementary Material Tables S10 and S11). Regarding all blood sampling times (n = 216), 82% were ≤ 3 min, 16% > 3 min, and for 2%, no data were obtained due to missing video recordings.

In total, only 29.6% of obtained CORT concentrations showed elevations (n = 64). Out of these, 65.6% were associated with a collection time ≤ 3 min, including 4.7% within 1 min and 31.3% with a time span > 3 min (Supplementary Material Table S2). Due to missing video recordings of the blood sampling, 3.1% of the CORT peaks could not be associated with the time span of blood sampling. From CORT peaks with a collection time ≤ 3 min, 33 peaks (78.6%) were not related to previous distress < 30 min before sampling, four peaks (9.5%) were associated with previous distress < 30 min before sampling, and five peaks (11.9%) could not be clearly assigned to previous distress or distress that occurred > 30 min before sampling. For 20 CORT peaks (9.2%), a time span of four to seven minutes was required due to problems with blood collection. Six of these elevations (2.7%) were clearly assigned to distress in the hens, including a sampling time of seven minutes, which only appeared once. The remaining 14 peaks, associated with a duration of four to six minutes (6.5%), were not related to obvious distress noticeable by video and audio recordings. A total of 15 blood samples (6.9%) did not show elevated CORT concentrations but were associated with sampling times of four to six minutes.

With regard to oviposition, exact time points for individual hens could not be determined, since monitoring of the laying nests was not possible, and similar plumage color and vests hindered differentiation of the individual hens. But, relating the nearest CORT peaks to the observed oviposition of all hens per day (between 06:14 a.m. and 04:56 p.m., as described above), the CORT peaks were estimated to occur 2 h 55 min to 10 h 12 min before oviposition.

No correlation was found for CORT peaks vs. aggressive behavior, as no aggression with harming was detected during the experiments. In 15.6% of the peaks, the increase in CORT was associated with exposure to dominance behavior as observed in the video analysis. An association with dominance behavior was uncertain for a further 7.8% of the CORT peaks. In these cases, a peak could not conclusively be assigned to dominance because either such behavior was not unequivocally visible on the videos, or it happened more than 30 min before blood sampling. The remaining 76.6% of CORT peaks were not associated with any observed dominant behavior.

In summary, most CORT peaks were not associated with overt behavioral events, neither were they due to shortcomings of the blood sampling.

## 4. Discussion

Under the given keeping conditions, the grouped CORT concentrations of all hens showed a significant peak between 12:00 p.m. and 04:00 p.m. (*p* < 0.05), and a furthrer noticeable although non-significant peak at 12:00 a.m. (0.12 < *p* < 0.53) (Figure 1). As all groups were kept under natural light through the daylight windows during the experiments, the duration of photophase varied between groups: 15L:9D (July, group one), 14L:10D (August, group two) and 11L:13D (September/October, group three). Regardless of the duration of photophase, every group showed one statistically significant CORT peak or at least a tendency for a CORT peak. All groups showed pronounced peaks during night and day when looking at the individual hens’ CORT concentrations (Figure 2, Figure 3 and Figure 4). In groups one and three, major peaks of CORT concentrations were inconsistent with the time of day, but in most cases, they occurred either between 12:00 a.m. and 04:00 a.m. or between 12:00 p.m. and 04:00 p.m. In contrast, most hens of group two showed peaks at 04:00 a.m. but also often at 08:00 p.m. Other time points showed individually different peaks but no general pattern. In most cases, individual peaks occurred at intervals of about 12 h, however, there were some interindividual variations. Major peaks showed up in intervals of 24 h. When the individual time span between the CORT peaks differed from 12 h, the following daily patterns were shifted.

Summing up, despite distinct individual variations in the time of the CORT peaks among the hens, most peaks occurred between 12:00 p.m. and 04:00 p.m. and between 12:00 a.m. and 04:00 a.m. and in intervals of 12 h.

Absolute CORT measures in domestic chickens differ heavily among studies [26,27,28,29,30,31,55]. Summing up, basal CORT concentrations have been described to range between 270 pg/mL and 2700 pg/mL [26,27,28,29,30,31,55]. The peak concentrations were reported to amount to 800 pg/mL to 6300 pg/mL [27,28,29,30,31,55]. The CORT concentrations obtained here were generally within this range.

Reasons for the circadian rhythm of CORT concentrations showing major peaks in 24 h intervals can only be speculated. One possible explanation is that circadian patterns of hormone activities or concentrations are controlled by the duration of photophase, as has been shown in other investigations [56]. The secretion of CORT is regulated by the hypothalamic–pituitary–adrenal axis (HPA-axis) via the adrenocorticotrophic hormone (ACTH) and local adrenocortical catecholamine secretion [57,58]. Considering the influence of light on CORT production, an increased photoperiod under stable conditions is able to stimulate gonadotropine-releasing hormone (GnRH) secretion, which, in turn, enhances steroid production [59]. So far, two types of GnRH have been described: cGnRH-I (Chicken GnRH-I) und cGnRH-II (Chicken GnRH-II) [60,61,62]. Melatonin, in contrast, regulates the release of the gonadotropine inhibitory hormone (GnIH), which depresses steroid secretion and, therefore, is a natural antagonist of CORT [59]. However, our observations show no alterations in the egg laying rate of the three groups under varying photoperiod from 11 to 15 h scotophase, with the exception of the non-laying hen ten. The rise in CORT seen between midday and the afternoon during our experiments could be a result of a melatonin decrease [63]. It is known that constant light exposition during early age can suppress melatonin secretion and disrupt circadian rhythm, accompanied by elevated CORT [64]. Low melatonin concentrations, in turn, are related to depression and distressed behavior [65]. High light intensity (≥30 Lux) increases severe feather pecking and mortality, and, therefore, distress, in hens [66]. In comparison to artificial light, natural light has been reported to decrease serum CORT concentrations, which seems to be related to ultraviolet light [67,68]. Investigations in turkeys which also belong to the order Galliformes revealed a pattern with two CORT peaks per day under a 14L:10D light regime [69], although measured in different animals. A major CORT peak occurred at the beginning or middle of photophase and a second minor one at scotophase.

Revealing underlying mechanisms of circadian rhythm such as the impact of GnRH or melatonin was not the subject of this study but the influence of light programs, natural and artificial lighting and light intensities as well as avian perception of light intensity (gallilux) should be part of subsequent studies, where concentrations of CORT, GnRH and melatonin should be measured as well. Combining the analysis of many possible influencing factors would have required many more animals. We chose to focus on the main question of a possible circadian CORT rhythm to reduce the number of animals (n = 12) according to the 3R principle for gaining a first insight. During our study, all animals were successively sheltered in the same aviary, handled by the same experimenter and were kept under natural light through the daylight windows to minimize the effect of the environment (such as air flow rate, angle of light incidence, artificial light, equipment of the aviary, stocking density or change of the experimenter) on our results.

Periodically occurring stressors might represent a second possible cause for the fluctuations in the CORT concentrations in the hens. Stressors might include a struggle for resources such as feed and water after oviposition, which could explain the higher CORT concentrations between midday and afternoon, matching a pattern described in an earlier study in broilers [30] as well as the periodical blood sampling.

We did not take the social hierarchy/status of each individual into account, as their similar plumage color and High-Visual chicken jackets hindered the distinction of the individuals. Miniature microphones were placed on every chicken’s back to differentiate possible distress calls of the individuals. Only two hens (hens two and five) had a lighter plumage color as subjectively perceived by the investigator, and, therefore, could be fitted into hierarchy. Vocal recordings of individuals did not allow for any conclusions to be drawn about the social status of every hen, as hierarchy was mostly maintained by dominance behavior without fighting and vocalizations. Hens two and five, both with a plumage with a brighter color of brown than their mates and obviously a low position in the hierarchy, were each subdued by their flock mates. Their lighter plumage color which led to an easy identification in the video recordings might have encouraged pecking and threatening, since these hens differed phenotypically from the others who had a dark brown plumage. Although both hens with light brown plumage experienced up to 20 pecking instances and 28 chasing instances, they did not consistently have the highest CORT values of the group, and their CORT increases were not always associated with previously experienced dominance behavior (Table 2, Supplementary Material Tables S2 and S4).

However, due to the speed of blood sampling and the catching of the hens at a short time interval, these potential stressors are unlikely to have caused the CORT rise between midday and afternoon. In particular, these events were the same at all sampling points. It has been described before that CORT increases occurred 45 s to 3 min after the start of blood sampling [16,17,18]. In our investigation, the mean time span from the start of catching to the end of blood sampling was 2.5 min, and there was no positive association between the duration of the sampling time and CORT concentrations. Overall, 82% of all blood samplings and 65.6% of the CORT peaks that occurred were associated with a sampling time (from catch to finished blood sampling) of ≤3 min. Nonetheless, 14 CORT peaks with a sampling time of four to six minutes (6.5%) were not related to obvious distress noticeable by video and audio recordings and, therefore, might be a result of prolonged handling for blood sampling. As 15 blood samples (6.9%) associated with sampling times of four to six minutes did not show elevated CORT concentrations, we cannot conclude that prolonged handling causes CORT elevations in our study. These results demonstrate that our training procedure presumably was sufficient to reduce stress responses in the hens [38]. Thus, our CORT values measured in the blood appear to reflect physiological fluctuations.

Video and audio recordings within our study did not prove the thesis that blood CORT elevations are mainly influenced by social interactions, especially by aggressive behavior of the hens, since only dominance but no aggression according to our definition was noticeable. Barely visible gestures such as eye contact or head position may have a significant impact on social interactions and associated distress [32]. When all hens were regarded, only about 15.6% of the documented CORT increases were associated with visually recognizable dominance behavior.

Regarding the role of elevated CORT concentrations during daytime, we can only speculate. Apart from their importance in organisms’ stress reactions, glucocorticoids like CORT have numerous important functions, showing a catabolic effect on proteins and increasing gluconeogenesis from amino acids as well as glycogenolysis in the liver, which leads to an increase in glucose concentration in the blood [14]. Furthermore, CORT increases fatty acid synthesis in the liver [70]. Immunosuppression may occur due to reduced protein biosynthesis caused by increased CORT concentrations, which leads to lower antibody production and suppression of cellular defense [7,13].

The role of CORT in reproduction has not yet been clearly defined. On the one hand, there are studies that indicate a negative influence of CORT on breeding behavior in favor of foraging [71,72]. Other studies show that increased plasma CORT concentrations increase foraging in parents and, consequently, lead to increased body weight in chicks, providing a fitness advantage [73]. However, several studies are consistent with increased food intake and foraging behavior in birds with high CORT concentrations [73,74,75]. The laying hens in our experiment did not show any signs of incubating the laid eggs during the four-hour time slot before we removed the eggs. Research on parenting behavior was not possible as no offspring was produced. We did not look explicitly at the food intake. But in general, food intake was not noticeably increased in comparison to the time before the experimental procedure.

The impact of CORT on the ovaries and ovulation is unclear so far, as both excitatory and inhibitory effects have been described [15]. But the act of oviposition itself seems to play a role in the elevation of CORT, and it is suggested that estrogen, progesterone and the luteinizing hormone (LH) with its peak likewise have a crucial impact on the elevation of CORT after oviposition [76,77,78,79]. The LH peak as well as the action of mesotocin and arginine vasotocin promote muscle contractions in the oviduct, required for oviposition [80]. An inhibition of the secretion of LH may be induced by permanently increased blood CORT concentrations, in turn causing lower sex steroid concentrations, and thus affecting further egg formation and oviposition [81,82]. In contrast, Etches (1979) described a circadian CORT rhythm with a CORT increase during scotophase in White Leghorn hens which appeared to be independent of ovulation [55]. Hence, we did not expect the circadian rhythm in chickens to be strongly influenced by ovulation. Nevertheless, as injections of CORT are able to induce ovulation and CORT concentrations appear to be regulated by a circadian rhythm, there is some evidence for a relation between ovulation and circadian CORT rhythm, but the underlying mechanisms are not clarified yet [55,83,84].

In our study, oviposition occurred daily in 11 out of 12 laying hens during the experiment, but video recordings did not allow us to observe the exact time. It is remarkable that hen ten, the only hen which did not lay any eggs during the experimental phase, at the same time displayed the lowest CORT values of all hens with nearly no fluctuations. Since this hen had not laid any eggs before the start of the experiment, it can be deduced that she was not yet in a reproductive state at the time the experiment was carried out. Suppressive effects of CORT on oviposition in our study are unlikely because no direct effects of CORT could be proved in hen ten or the other hens as we would have expected.

Further study should be performed to examine the correlation of CORT with egg production and oviposition where estrogen, progesterone and LH as well as mesotocin and arginine vasotocin should be measured additionally to CORT under video documentation of oviposition time and labelling of the individuals.

The results obtained in our study serve as a basis for further research. Regarding practical relevance in veterinary medicine, our results can be used as an additional tool for distress assessment. CORT values are not a stand-alone indicator for distress since a single value does not provide information about basal or actual stress-related CORT values. Considering CORT daily fluctuations, one should take a minimum of two blood samples for CORT measurement when not combining distress assessment with other blood-related measurements such as the H:L ratio, leucocyte count or blood chemical parameters. For this, we would recommend taking samples in the morning within the first two hours after dawn and in the late afternoon in order to obtain non-elevated CORT concentrations and calculating the average CORT concentration. As a result, the influence of individual physiological fluctuations can be reduced, and the significance is increased. Behavioral observations or determination of the H:L ratio (elevation by stress) [10,27,85,86] may support the diagnosis of distress-related CORT elevation, whereas measurement of CORT concentrations might help to distinguish stress-related (high CORT) from infection-related (low CORT) leucocytosis in the blood [87,88].

Blood chemical parameters may be altered by elevated CORT concentrations which may occur due to prolonged distress. Elevated glucose values in chickens (reference 227–300 mg/dL) can be found as a result of increased gluconeogenesis and glycogenolysis [14,89,90]. Furthermore, creatinine values are higher in prolonged distress periods than physiological reference values which can be determined by laboratory diagnostics (reference 0.9–1.8 mg/dL) [90,91]. Changes in lipid metabolism lead to steatosis hepatitis in chickens and may result in elevated serum bile acids [91,92,93,94].

For the application of CORT measurement in practice, commercial ELISA kits can be used instead of a time-consuming and expensive measurement using radioimmunoassay. However, only a few of them (e.g., Corticosterone ELISA kit, Enzo Life Sciences, Inc., Farmingdale, NY, USA) are suitable or validated for the measurement of avian CORT [95].

## 5. Conclusions

This study reveals a circadian rhythm of blood CORT concentrations in domestic chickens with clear individual variations. Throughout the day, blood CORT concentrations in laying hens show two peaks, with CORT concentrations most probably regulated by photophase but possibly also by social interactions and reproduction, whereby statistically, only the major peak between 12:00 p.m. and 04:00 p.m. is significant (*p* < 0.05). The second tendency for a peak occurs at 12:00 a.m. (0.12 < *p* < 0.53). In most cases, individual peaks occurred in intervals of about 12 h; however, there were some interindividual variations. Major peaks showed up in intervals of 24 h. Further studies are necessary to investigate the reproducibility of our results, seasonal and stressor type influences, the influence of light programs, natural and artificial lighting, light intensities as well as avian perception of light intensity (gallilux), while measuring blood concentrations of CORT, GnRH and melatonin. A complementary study should examine the correlation of CORT with egg formation and oviposition where estrogen, progesterone and LH, as well as mesotocin and arginine vasotocin have to be determined additionally to CORT, adding video documentation of oviposition time and labelling of the individuals. In our study, we exclusively focused on the verification of a circadian CORT rhythm in laying hens under social interactions. Our results proved the presence of a circadian CORT rhythm in chickens and created a basis for further basic research.

## Figures and Tables

**Table 1:** Blood sampling table for each test series.

Day	Sampling Point	Time
1	1	12:00 p.m.
	2	04:00 p.m.
	3	08:00 p.m.
	4	12:00 a.m.
	5	04:00 a.m.
	6	08:00 a.m.
2	7	12:00 p.m.
	8	04:00 p.m.
	9	08:00 p.m.
	10	12:00 a.m.
	11	04:00 a.m.
	12	08:00 a.m.
3	13	12:00 p.m.
	14	04:00 p.m.
	15	08:00 p.m.
	16	12:00 a.m.
	17	04:00 a.m.
	18	08:00 a.m.

**Table 2:** Number of experienced dominance behavior and blood sampling related distress per hen.

	Being Pecked (Audio and Video)		Being Chased (Audio and Video)		Blood Sampling-Related Distress during a Test Series (Audio and Video)	
Hen	Total/Day	Mean/Day	Total/Day	Mean/Day	Total	Mean/Day
01	0		0		0	
02	0–26	14.8	1–28	18	0	
03	0–2	0.3	0		4	1.3
04	0–4	1.3	0–1	0.2	1	0.3
05	0–7	3.0	0–2	0.8	1	0.3
06	0–1	0.3	0		1	0.3
07	0–2	0.5	0–1	0.2	0	
08	0–1	0.3	0		0	
09	0–2	0.5	0		5	1.7
10	0		0		2	0.7
11	0–1	0.2	0		1	0.3
12	0–1	0.2	0		0	

## Data Availability

Data are contained within the article and Supplementary Materials.

## Supplementary Materials

The following supporting information can be downloaded at: https://www.mdpi.com/article/10.3390/ani14060873/s1. Table S1: Information on the experimental phase. Table S2: Observations at corticosterone peaks. Table S3: Summarized blood corticosterone measurements (pg/mL), descriptive statistics of all hens for each time point, excl. first measurement of each hen. Table S4: Total blood corticosterone measurements (pg/mL) of all hens for all sampling points. Table S5: Blood corticosterone measurements (pg/mL), descriptive statistics for each hen, excl. first measurement of each hen. Table S6: Shapiro-Wilk-Test for summarized blood corticosterone measurements of all hens for each time point, excl. first measurement of each hen. Table S7: Robust linear mixed model fit by DAStau for summarized blood corticosterone measurements (pg/mL) of all hens for each time point, excl. first measurement of each hen. Table S8: Estimated marginal means (emmeans) of summarized blood corticosterone measurements (pg/mL) of all hens for each time point, excl. first measurement of each hen, confidence level used: 0.95. Table S9: Contrasts of emmeans of summarized blood cortiocosterone measurements (pg/mL) of all hens for each time point, excl. first measurement of each hen, confidence level used: 0.95. Table S10: Correlation amount disstress-related peaks with blood sampling time. Table S11: Pearson’s Chi-squared test, Correlation disstress-related peaks with blood sampling time. Figure S1: Spectrograms of call types: (a) food calls, (b) gakel calls, (c) contentment call, (d) short scream, (e) long scream, (f) growling, (g) whining, (h) mixed call, (i) single cluck, (j) double cluck, (k) fast clucks. Figure S2: Q-Q plot for total corticosterone measurements, excl. first measurement of each hen. Figure S3: Kruskal Wallis rank sum test for Corticosterone measurements of all hens, excl. first measurement of each hen. Figure S4: Corticosterone effect plot (pg/mL) for hens 01 to 04, excl. first measurement of each hen. Figure S5: Corticosterone effect plot (pg/mL) for hens 05 to 08, excl. first measurement of each hen. Figure S6: Corticosterone effect plot (pg/mL) for hens 09 to 12, excl. first measurement of each hen.
