# Supplementary material for: Circadian Corticosterone Profile in Laying Hens (Gallus gallus domesticus)
Source: Animals (Basel). 2024 Mar 12;14(6):873. doi: 10.3390/ani14060873 (PMC10967282; doi:10.3390/ani14060873)
Supplement: Supplementary file 1 [file animals-14-00873-s001.zip › animals-2864721-supplementary.pdf]

Supplementary Material

Table S 1. Information on the experimental phase

Group	Test series	Date	Tested hens	Remarks
**1**	1	2021/07/21-24	3 and 4	Problems with microphone recordings,<br>07/23 from 10.38 am to 12.23 pm no video recording because of insufficient storage space
	2	2021/07/28-31	1 and 2	Problems with microphone recordings, 07/28-30 hen 1 audio not recorded -> change of microphone for hen 1 at 07/30, 12.35 pm
**2**	3	2021/08/12-15	6 and 8	Installation of additional external microphone in the aviary to supplement audio recordings in case of failing
	4	2021/08/18-21	5 and 7	/
**3**	5	2021/09/29-10/02	11 and 12	/
	6	2021/10/06-09	9 and 10	/

Table S 2. Observations at corticosterone peaks

Hen (number)	***sampling point (number)***	***date***	***time***	***CORT peaks associated with dominance behavior or distress***			***time span catch to blood sampling (minutes)***	***time span between hormone peaks (hrs.min)***
				Yes (< 30 minutes before blood sampling)	no	not clearly visible or > 30 minutes prior blood sampling		
1	1	21/07/28	12.07 pm		x		no recording	15.55
	5	21/07/29	04.02 am		x		6	11.56
	8	21/07/29	03.58 pm		x		4	11.58

	11	21/07/30	03.56 am		x		2	12.01
	14	21/07/30	03.57 pm		x		3	8.03
	16	21/07/31	12.00 am		x		2	7.58
	18	21/07/31	07.58 am		x		2	
2	1	21/07/28	12:11 pm		x		no recording	23.52
	7	21/07/29	12:03 pm	x (repeatedly being pecked and chased from food, 20x between 08.00 am and 12.00 pm)			5	12.04
	10	21/07/30	12.07 am		x		5	12.01
	13	21/07/30	12.08 pm	x (repeatedly being pecked and chased from food, 16x between 07.40 am and 11.30 am)			6	
3	2	21/07/21	04.02 pm		x		4	16.06
	6	21/07/22	08.08 am		x		4	15.49
	10	21/07/22	11.57 pm	x (caught for blood sampling)			5	16.01
	14	21/07/23	03.58 pm		x		3	11.59
	17	21/07/24	03.57 am	x (caught for blood sampling)			2	
4	2	21/07/21	04.12 pm			x (stress call 4 h ago)	3	11.49
	5	21/07/22	04.01 am		x		4	7.54
	7	21/07/22	11.55 am			x (2x being pecked at 09.41 am and 10.31 am)	2	36.04
	16	21/07/23	11.59 pm	x (caught for blood sampling)			4	
5	1	21/08/18	11.54 am		x		2	16.04
	5	21/08/19	03.58 am		x		3	8.00
	7	21/08/19	11.58 am	x (10.25 am chased from food)			2	8.00
	9	21/08/19	07.58 pm		x		2	8.03
	11	21/08/20	04.01 am		x		5	15.57
	15	21/08/20	07.58 pm			x	3	12.02

						(4x being pecked between 09.10 am and 05.29 pm)		
	18	21/08/21	08.00 am		x		1	
6	2	21/08/12	03.54 pm		x		2	12.05
	5	21/08/13	03.59 am		x		6	16.00
	9	21/08/13	07.59 pm		x		4	12.01
	12	21/08/14	08.00 am		x		2	19.58
	17	21/08/15	03.58 am		x		3	
7	1	21/08/18	11.57 am		x		1	12.06
	4	21/08/19	12.03 am		x		2	20.02
	9	21/08/19	08.05 pm		x		3	15.57
	13	21/08/20	12.02 pm			x (2x being pecked/chased at 09.39 am and 10.54 am)	3	16.02
	17	21/08/21	04.04 am		x		2	
8	1	21/08/12	12.01 pm		x		2	20.13
	6	21/08/13	08.14 am		x		3	19.48
	11	21/08/14	04.02 am		x		2	12.02
	14	21/08/14	04.04 pm			x (03.15/03.16 pm being pecked)	2	7.58
	16	21/08/15	12.02 am		x		2	8.02
	18	21/08/15	08.04 am		x		2	
9	2	21/10/06	03.57 pm		x		2	12.04
	5	21/10/07	04.01 am	x (caught for blood sampling)			7	11.53
	8	21/10/07	03.54 pm		x		6	20.04
	13	21/10/08	11.58 am		x		2	7.58
	15	21/10/08	07.56 pm	x (caught for blood sampling)			2	
10	1	21/10/06	12.07 pm		x		2	11.58
	4	21/10/07	12.05 am		x		3	55.55
	18	21/10/09	08.00 am	x (caught for blood sampling)			2	
11	1	21/09/29	12.04 pm	x			4	11.49

				(11.08 am fallen from ventilation system)			
	4	21/09/29	11.53 pm		x	2	12.07
	7	21/09/30	12.00 pm		x	2	11.56
	10	21/09/30	11.56 pm		x	4	12.04
	13	21/10/01	12.00 pm		x	4	12.03
	16	21/10/02	12.03 am		x	5	
12	1	21/09/29	12.13 pm		x	3	15.44
	5	21/09/30	03.57 am		x	2	8.06
	7	21/09/30	12.03 pm		x	1	8.01
	9	21/09/30	08.04 pm		x	5	12.01
	12	21/10/01	08.05 am		x	2	7.58
	14	21/10/01	04.03 pm		x	3	11.59
	17	21/10/02	04.02 am		x	2	

Table S 3. Summarized blood corticosterone measurements (pg/ml), descriptive statistics of all hens for each time point, excl. first measurement of each hen

	**12.00 am**	**04.00 am**	**08.00 am**	**12.00 pm**	**04.00 pm**	**08.00 pm**
**Min.**	530.1	485.3	479.3	486.8	504.6	494
**1st Qu.**	1210.9	1293.6	1172.4	946.4	1116	726.7
**Median**	2330.4	2083	1719.8	2661.6	2791.3	1737.1
**Mean**	2628	2469.4	1975.2	3101.1	3030.5	2241
**3rd Qu.**	3556.5	2684.5	2520.8	4581.4	4776.4	2966.7
**Max.**	7950.8	9593.6	4919.6	9958.9	7522.5	8163.5
**SD**	1852	1957.5	1161.2	2467.3	2112.7	1921.8

Table S 4. Total blood corticosterone measurements (pg/ml) of all hens for all sampling points

Sampling Point	Hen 01	Hen 02	Hen 03	Hen 04	Hen 05	Hen 06	Hen 07	Hen 08	Hen 09	Hen 10	Hen 11	Hen 12
**1 (12.00 pm)**	11337.88	37068.27	2307.67	2335.83	3336.32	1995.83	4404.87	8782.14	3994.85	9788.77	7800.25	12106.48
**2 (04.00 pm)**	5095.93	6651.09	3963.98	3264.00	1861.96	5698.87	779.25	568.70	4722.59	3181.24	1393.52	7522.46
**3 (08.00 pm)**	7630.30	3290.02	2764.11	1870.20	526.28	3469.83	622.02	522.23	2837.19	493.97	1603.92	4774.27
**4 (12.00 am)**	7950.79	1422.17	3311.44	2493.69	1646.19	4297.83	2350.36	706.56	1219.75	1184.26	3049.51	3538.03
**5 (04.00 am)**	8014.69	2087.32	4024.38	4165.21	2319.44	5200.75	1305.51	1161.32	4224.67	490.12	1491.80	4351.45
**6 (08.00 am)**	1422.68	4919.63	4181.82	3014.24	716.10	4268.71	1224.65	3975.19	1654.56	479.26	1485.59	2394.46
**7 (12.00 pm)**	1917.62	5563.41	3011.64	9958.90	939.27	2311.54	514.94	3200.94	3468.19	486.78	5529.48	5739.68
**8 (04.00 pm)**	5251.65	5050.30	2886.05	4208.96	529.37	2892.52	813.75	1069.49	4023.79	504.59	1094.46	1844.51
**9 (08.00 pm)**	571.23	3616.93	1057.69	2246.24	1986.00	5623.81	3849.95	531.61	3370.76	498.45	1213.69	8163.50
**10 (12.00 am)**	6087.10	6193.54	4925.26	2394.54	1232.09	3611.79	530.07	998.15	2872.99	547.99	5195.56	2310.52
**11 (04.00 am)**	9593.64	1512.50	2466.94	2278.06	2186.01	940.52	515.66	2683.63	1804.58	485.27	1897.98	2449.77
**12 (08.00 am)**	2901.22	1610.55	1474.27	2254.15	1840.60	1947.31	2009.66	2492.91	1001.00	507.69	872.75	3574.64
**13 (12.00 pm)**	3236.69	6990.52	1434.59	1780.71	948.77	1947.37	4265.40	519.34	3536.34	529.36	5862.60	732.78
**14 (04.00 pm)**	4937.92	6844.06	2696.47	1822.47	539.29	1740.00	1353.55	2251.54	1123.18	573.67	3636.83	6705.21
**15 (08.00 pm)**	2858.89	2769.02	620.16	1969.05	1155.55	957.40	761.56	523.96	1562.09	918.35	2367.78	1077.83
**16 (12.00 am)**	3437.97	1485.78	2385.01	3653.27	852.16	1638.99	1374.29	1159.98	858.71	920.72	5384.59	1386.98
**17 (04.00 am)**	2686.96	1257.69	3322.05	2204.42	1117.57	2314.46	1787.44	753.24	517.85	1329.65	1876.19	2078.60
**18 (08.00 am)**	3460.48	1015.70	2604.49	1237.26	1785.03	1345.90	582.68	1826.50	554.61	1926.23	998.68	1544.89

Table S 5. Blood corticosterone measurements (pg/ml), descriptive statistics for each hen, excl. first measurement of each hen

	Hen 01	Hen 02	Hen 03	Hen 04	Hen 05	Hen 06	Hen 07	Hen 08	Hen 09	Hen 10	Hen 11	Hen 12
**Min.**	571.2	1016	620.2	1237	526.3	940.5	514.9	519.3	517.9	479.3	872.7	732.8
**1st Qu.**	2858.9	1513	2385	1969	852.2	1740	622	568.7	1123.2	494	1393.5	1844.5
**Median**	3460.5	3290	2764.1	2278	1155.5	2314.5	1224.7	1069.5	1804.6	529.4	1876.2	2449.8
**Mean**	4532.7	3664	2772.4	2989	1304.8	2953.4	1449.5	1467.4	2314.9	885.7	2644.4	3540.6
**3rd Qu.**	6087.1	5563	3322	3264	1840.6	4268.7	1787.4	2251.5	3468.2	920.7	3636.8	4774.3
**Max.**	9593.6	6991	4925.3	9959	2319.4	5698.9	4265.4	3975.2	4722.6	3181.2	5862.6	8163.5
**SD**	2592.4	2214.4	1161.8	1983.7	606.3	1593.4	1128.3	1077.9	1390.7	716.4	1779.8	2316.5

Table S 6. Shapiro-Wilk-Test for summarized blood corticosterone measurements of all hens for each time point, excl. first measurement of each hen

Time point	W	p-value
$’12.00 am‘	0.88898	0.001728
$’04.00 am‘	0.78671	8.963e-06
$’08.00 am‘	0.9201	0.01267
$’12.00 pm‘	0.89295	0.0153
$’04.00 pm‘	0.91833	0.01125
$’08.00 pm‘	0.82316	4.863e-05

Table S 7. Robust linear mixed model fit by DAStau for summarized blood corticosterone measurements (pg/ml) of all hens for each time point, excl. first measurement of each hen

Formula: value ~ time + (1 | Hen)

Data: CORT					
Random effects:					
**Groups**	**Name**	**Std.Dev.**			
Hen	(Intercept)	1016			
Residual	1484				
Number of obs: 204, groups: Hen, 12					
**Fixed Effects:**					
**(Intercept)**	**time 2(04.00 am)**	**time 3(08.00 am)**	**time 4(12.00 pm)**	**time 5(04.00 pm)**	**time 6(08.00 pm)**
2498.4	-223.3	-557.3	287.9	375.5	-446.1

Table S 8. Estimated marginal means (emmeans) of summarized blood corticosterone measurements (pg/ml) of all hens for each time point, excl. first measurement of each hen, confidence level used: 0.95

**time**	**emmean**	**SE**	**df**	**asymp. LCL**	**asymp. UCL**
**1 (12.00 am)**	2498	393	Inf	1727	3270
**2 (04.00 am)**	2275	393	Inf	1504	3046
**3 (08.00 am)**	1941	393	Inf	1170	2712
**4 (12.00 pm)**	2786	432	Inf	1939	3634
**5 (04.00 pm)**	2874	393	Inf	2103	3645
**6 (08.00 pm)**	2052	393	Inf	1281	2824

Table S 9. Contrasts of emmeans of summarized blood cortiocosterone measurements (pg/ml) of all hens for each time point, excl. first measurement of each hen, confidence level used: 0.95

contrast	estimate	SE	df	z.ratio	p.value
**1 (12.00 am) - 2 (04.00 am)**	223.3	359	Inf	0.623	0.5336
**1 (12.00 am) - 3 (08.00 am)**	557.3	359	Inf	1.554	0.1202
**1 (12.00 am) - 4 (12.00 pm)**	-287.9	401	Inf	-0.718	0.4728
**1 (12.00 am) - 5 (04.00 pm)**	-375.5	359	Inf	-1.047	0.2952
**1 (12.00 am) - 6 (08.00 pm)**	446.1	359	Inf	1.244	0.2135
**2 (04.00 am) - 3 (08.00 am)**	334	359	Inf	0.931	0.3517
**2 (04.00 am) - 4 (12.00 pm)**	-511.1	401	Inf	-1.275	0.2024
**2 (04.00 am) - 5 (04.00 pm)**	-598.7	359	Inf	-1.669	0.0951
**2 (04.00 am) - 6 (08.00 pm)**	222.9	359	Inf	0.621	0.5343
**3 (08.00 am) - 4 (12.00 pm)**	-845.2	401	Inf	-2.108	0.0351
**3 (08.00 am) - 5 (04.00 pm)**	-932.7	359	Inf	-2.601	0.0093
**3 (08.00 am) - 6 (08.00 pm)**	-111.1	359	Inf	-0.310	0.7566
**4 (12.00 pm) - 5 (04.00 pm)**	-87.6	401	Inf	-0.218	0.8271
**4 (12.00 pm) - 6 (08.00 pm)**	734	401	Inf	1.830	0.0672
**5 (04.00 pm) - 6 (08.00 pm)**	821.6	359	Inf	2.291	0.0220

Table S 10. Correlation amount disstress-related peaks with blood sampling time

	**Blood sampling time (min)**						
	1	2	3	4	5	6	7
**Distress**							
Yes	**0**	**4**	**0**	**2**	**2**	**1**	**1**
No	**3**	**21**	**9**	**7**	**4**	**3**	**0**
Uncertain	**0**	**2**	**3**	**0**	**0**	**0**	**0**

Table S 11. Pearson's Chi-squared test, Correlation disstress-related peaks with blood sampling time

X-squared	df	p-value
15.526	12	0.2139

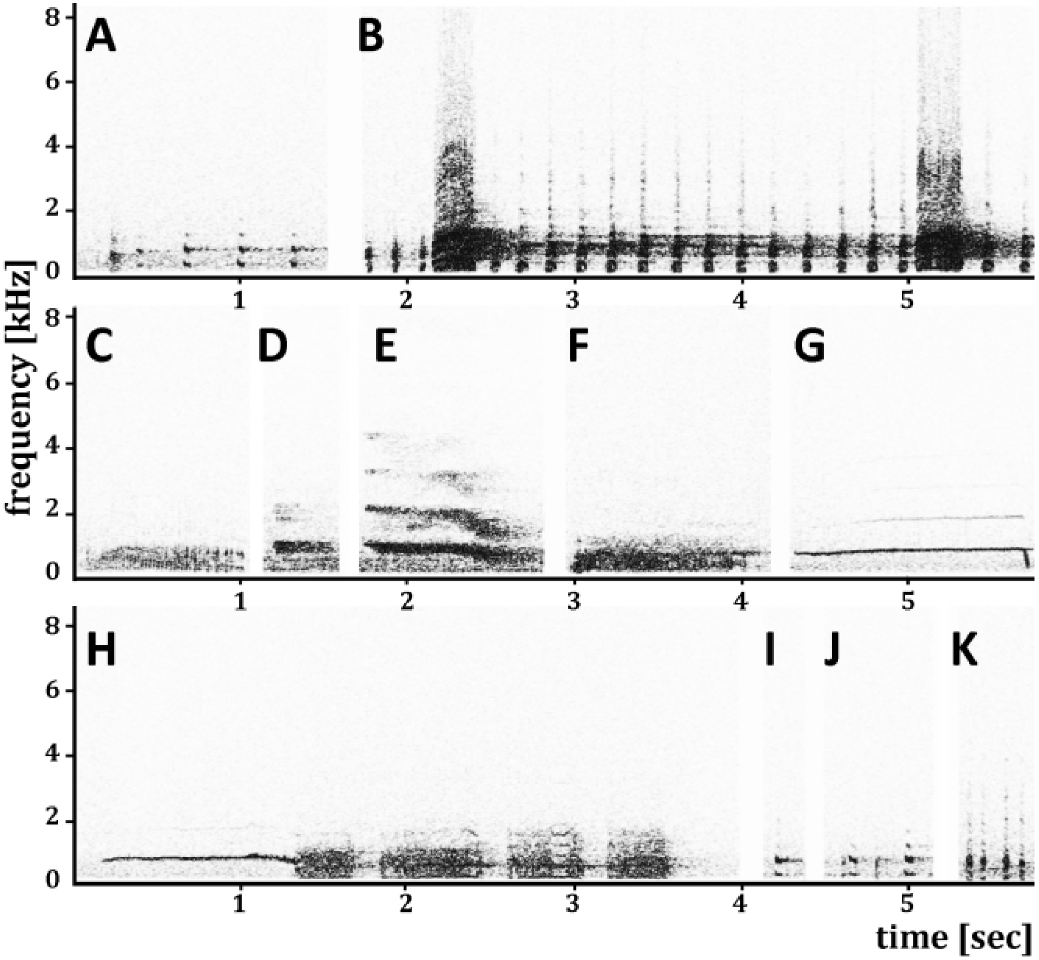

Figure S 1. Spectrograms of call types: a) food calls, b) gakel calls, c) contentment call, d) short scream, e) long scream, f) growling, g) whining, h) mixed call, i) single cluck, j) double cluck, k) fast clucks

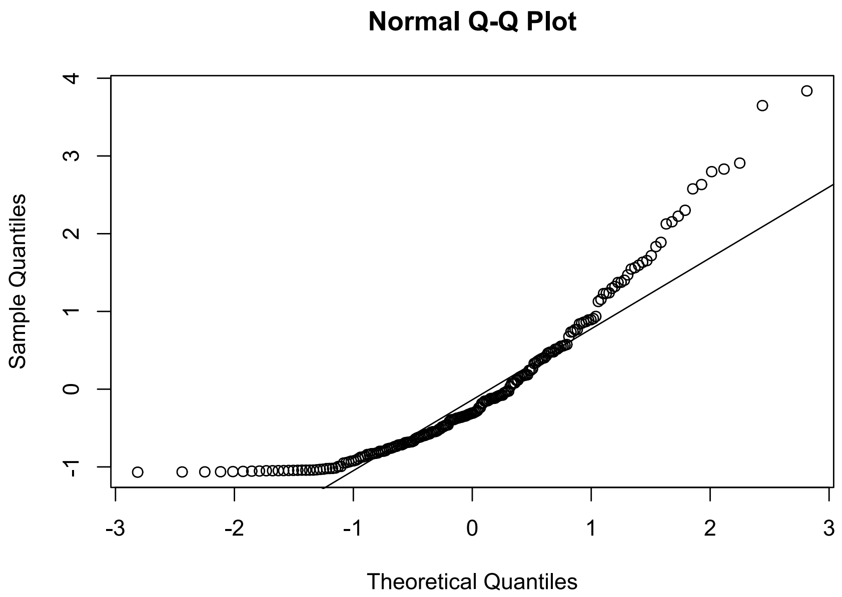

Figure S 2. Q-Q plot for total corticosterone measurements, excl. first measurement of each hen

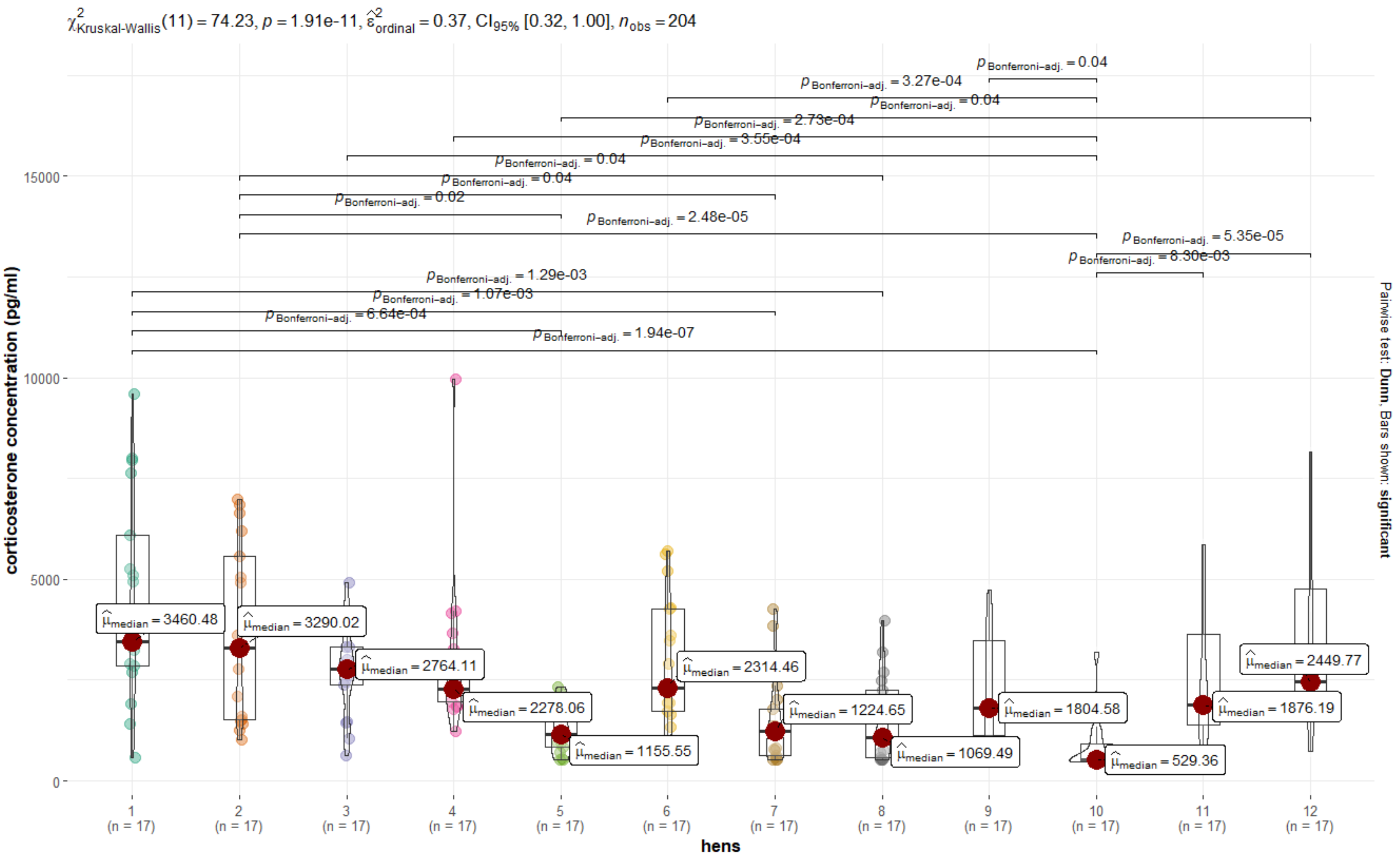

Figure S 3. Kruskal Wallis rank sum test for Corticosterone measurements of all hens, excl. first measurement of each hen

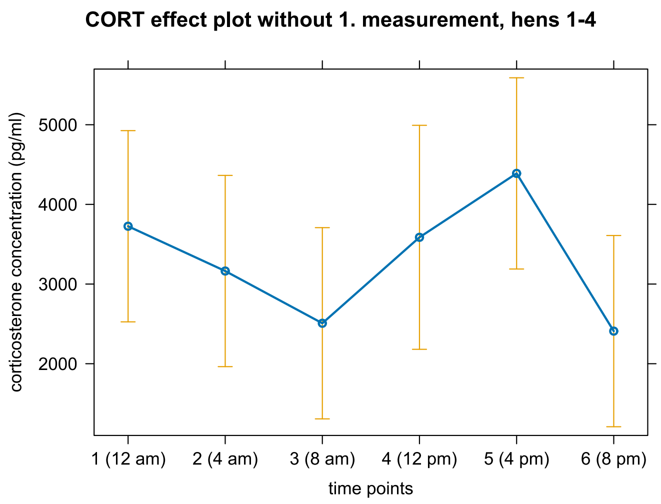

Figure S 4. Corticosterone effect plot (pg/ml) for hens 01 to 04, excl. first measurement of each hen

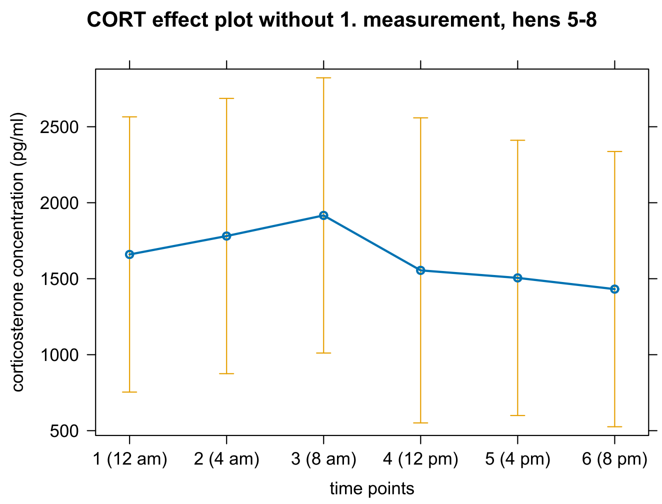

Figure S 5. Corticosterone effect plot (pg/ml) for hens 05 to 08, excl. first measurement of each hen

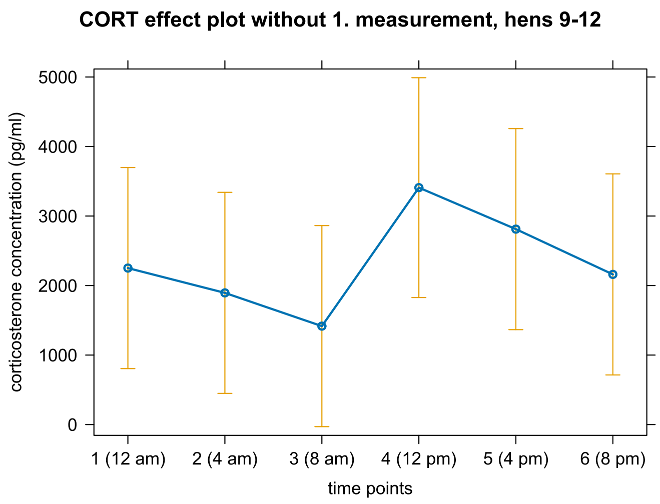

Figure S 6. Corticosterone effect plot (pg/ml) for hens 09 to 12, excl. first measurement of each hen
